# Prevalence of *Fusarium* fungi and Deoxynivalenol Levels in Winter Wheat Grain in Different Climatic Regions of Poland

**DOI:** 10.3390/toxins14020102

**Published:** 2022-01-28

**Authors:** Adam Okorski, Alina Milewska, Agnieszka Pszczółkowska, Krzysztof Karpiesiuk, Wojciech Kozera, Joanna Agnieszka Dąbrowska, Justyna Radwińska

**Affiliations:** 1 Department of Entomology, Phytopathology and Molecular Diagnostics, Faculty of Agriculture and Forestry, University of Warmia and Mazury in Olsztyn, Plac Łódzki 5, 10-727 Olsztyn, Poland; alina.charenska2209@gmail.com (A.M.); agnieszka.pszczolkowska@uwm.edu.pl (A.P.); joanna.dabrowska@uwm.edu.pl (J.A.D.); 2 Department of Pig Breeding, Faculty of Animal Bioengineering, University of Warmia and Mazury in Olsztyn, Oczapowskiego 5, 10-719 Olsztyn, Poland; krzysztof.karpiesiuk@uwm.edu.pl (K.K.); wojciech.kozera@uwm.edu.pl (W.K.); 3 Department of Internal Diseases with Clinic, Faculty of Veterinary Medicine, University of Warmia and Mazury in Olsztyn, Oczapowskiego 14, 10-718 Olsztyn, Poland; justyna.radwinska@uwm.edu.pl

**Keywords:** deoxynivalenol, *F. avenaceum*, *F. graminearum*, qPCR, competition, synergy, climate change

## Abstract

*Fusarium* head blight (FHB) caused by fungi of the genus *Fusarium* is one of the most dangerous crop diseases, which has a wide geographic distribution and causes severe economic losses in the production of major cereal species. The infection leads to the accumulation of mycotoxins in grains, which compromises its suitability for human and animal consumption. The study demonstrated that grain samples from warmer regions of Poland, including Sulejów and Tomaszów Bolesławicki (results differed across years of the study), were colonized mainly by *F. graminearum* and were most highly contaminated with deoxynivalenol (DON). Samples from Northeastern Poland, i.e., Ruska Wieś, which is located in a cooler region, were characterized by a predominance of *Fusarium* species typical of the cold climate, i.e., *Fusarium poae* and *Penicillium verrucosum*. A Spearman’s rank correlation analysis revealed that the severity of grain infection with *F. avenaceum*/*F. tricinctum* was affected by the mean daily temperature and high humidity in May, and the corresponding values of the correlation coefficient were determined at R = 0.54 and R = 0.50. Competitive interactions were observed between the *F. avenaceum*/*F. tricinctum* genotype and DON-producing *F. culmorum* and *F. graminearum*, because the severity of grain infections caused by these pathogens was bound by a negative correlation.

## 1. Introduction

Cereal crops are exposed to various types of environmental stress associated with the availability of water, light and minerals, as well as bacterial and fungal infections [1]. The rapid increase in area under winter wheat and the high share of cereals in the structure of arable land increase the severity of fungal diseases. Winter wheat is susceptible to many diseases that can decrease yields [2,3]. Cereal diseases caused by fungi of the genus *Fusarium* include seedling blight that leads to pre- and post-emergence damping off [4], *Fusarium* foot rot [5] and *Fusarium* head blight (FHB) [6]. On the global scale, FHB is considered the most dangerous disease of wheat that generates the greatest economic losses. Severe FHB outbreaks have been reported every 4 to 5 years in the USA, China, the European Union (EU), Great Britain, Africa and Brazil [7]. FHB is caused by fungi of the genus *Fusarium* [2], and *F. graminearum* is the most prevalent pathogen around the world [8,9]. In Poland, the predominant *Fusarium* pathogens of cereals, including winter wheat, include *F. avenaceum, F. crookwellense*, *F. culmorum, F. equiseti, F. graminearum, F. langsethiae, F. sporotrichioides, F. oxysporum, F. poae* and *F*. *tricinctum* [10,11,12,13].

Saprotrophic mycelia in crop residues, chlamydospores and conidia that are spread by wind, rain and insects in the flowering stage are the main sources of FHB infection in successive years [6,14]. Fungal spores germinate on the surface of spikelets, and the mycelium penetrates spikelets passively through the stomata or actively through the cell walls. *Fusarium* fungi secrete numerous hydrolyzing enzymes that facilitate the penetration of host tissues [15]. Infected kernels are smaller, shriveled and white to light pink in color [6].

Due to rapid industrial growth powered mainly by fossil fuels, carbon dioxide (CO_2_) concentrations in the atmosphere exceeded 409 µmol mol^−1^ in 2019, which could contribute to climate change [16]. Global climate change affects plant growth, photosynthetic parameters, crop yields and seed/grain quality parameters such as the protein content [17]. Increasing atmospheric CO_2_ levels influence the seed germination capacity; seed vigor; moisture content; health status and physical parameters such as size, shape, weight and color [18].

The territory of Poland covers several climatic regions that differ in temperature [19], ranging from the coldest 5B region in the northeast to the mildest 7B region in the coastal zone of Northwestern Poland. Climate change increases temperatures and decreases precipitation levels in Central Europe and in other regions of the world [20,21]. According to the latest research on the Polish climate, the coldest 5B region has nearly disappeared, whereas the milder 7A and 6B regions have been visibly expanded [22]. The severity of FHB is determined mainly by weather conditions in the flowering stage when wheat stands are most often infected [23]. Precipitation, humidity and temperature significantly influence the severity of the disease symptoms [24]. According to epidemiological studies, the incidence of FHB is determined by the presence of the inoculum during the growing season, sporulation rate, duration of the infectious stage and inoculum potential [25].

Fungi that cause FHB produce toxic metabolites that directly damage kernels and can be accumulated in stored grains [6]. Trichothecenes are an important group of mycotoxins produced by *Fusarium* fungi [26]. Trichothecenes have been divided into four groups (A, B, C and D) based on structural differences. Type A (T-2, HT-2, neosolaniol and diacetoxyscirpenol) and type B (deoxynivalenol—DON, DON derivatives, nivalenol and fusarin X) trichothecenes are ubiquitous in the environment [27]. Trichothecenes are the leading group of *Fusarium* mycotoxins that cause chronic or even lethal toxicoses in both humans and animals. These compounds exert toxic effects by inhibiting ribosomal protein synthesis. Trichothecenes are a large and diverse group of compounds, but T-2 mycotoxin, nivalenol (NIV), DON and diacetoxyscirpenol (DAS) are most frequently identified in cereal grains [28]. Deoxynivalenol is detected mainly in wheat and barley, and it is less frequently noted in oat and rye grains. On average, DON is identified in 57% of wheat samples worldwide [29]. The discussed compounds exert neurotoxic and teratogenic effects, and they can cause acute and chronic immune disorders in humans and animals [30]. According to the latest literature, new research programs are needed to address the impact of global climate change on the risk of FHB infections in cereals [31].

The present study was undertaken to explore this problem. The aim of this study was to determine the prevalence of DON-producing *Fusarium* fungi and the concentration of DON in wheat grains grown in different climatic regions of Poland and to describe the interactions between DON-producing fungi and other *Fusarium* species in the analyzed grains.

## 2. Results

### 2.1. Culture-Based Morphological Method for the Identification of Fungi

A total of 1598 fungal isolates belonging to 21 taxa and selected isolates classified as nonsporulating fungi were obtained from the grains of both wheat varieties during in the study (Table S1). The most prevalent species, *A. alternata* and *E. nigrum*, accounted for 69.77% and 17.02% of all the isolates, respectively. *Fusarium* fungi accounted for 6.07% of the examined isolates and were represented by eight taxa (*F. avenaceum, F. culmorum, F. equiseti, F. graminearum, F. oxysporum F. poae, F. sporotrichioides* and *F. tricinctum*), as well as individual isolates that were identified to the genus level only. In both years of the study, most *Fusarium* isolates were isolated from wheat grain in Ruska Wieś (Table S1 and Figure S1).

### 2.2. Quantification of F. culmorum and F. graminearum DNA by qPCR and Quantification of DON by HPLC

A multiple analysis of variance revealed that the level of grain infection by *Fusarium* fungi measured by the qPCR method was affected by location but not by wheat variety (Table 1).

Multiple analyses of variance showed that the level of grain infection by *Fusarium* fungi measured by the qPCR method depends on the location but not the variety of wheat.

**Figure 1:**
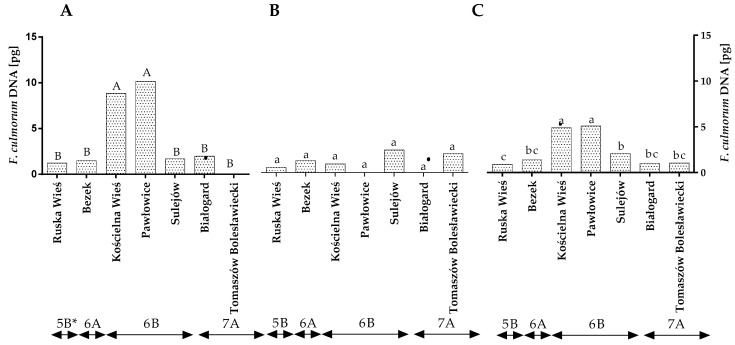
Quantity of *F. culmorum* DNA (pg) in winter wheat grains grown in different locations in Poland (* climatic regions) (**A**) during 2015, (**B**) during 2016 and (**C**) the average over the years of the study. a, b and c—significant at *p* ≤ 0.05; A, B and C—significant at *p ≤* 0.01.

The qPCR analysis revealed that the quantity of *F. culmorum* DNA varied across the years of the study (Figure 1A,B). In the first year, the highest amount of *F. culmorum* DNA was found in Pawłowice and Kościelna Wieś, and its amount was significantly lower in the remaining locations. Significant differences were found between locations in region 6B (Figure 1A,C and Table S2). In the second year, no differences in the quantity of *F. culmorum* DNA were noted between locations or climate regions (Figure 1B). The statistical analysis demonstrated that, during the study, the average quantity of *F. culmorum* DNA ranged from 0.99 pg in Ruska Wieś to 4.89 pg in Kościelna Wieś and 5.08 pg in Pawłowice (Figure 1C). The average quantity of *F. culmorum* DNA was the highest (4.01 pg) in winter wheat grains grown in climatic region 6B and lowest (0.9 pg) in region 5B (Figure 1C).

**Figure 2:**
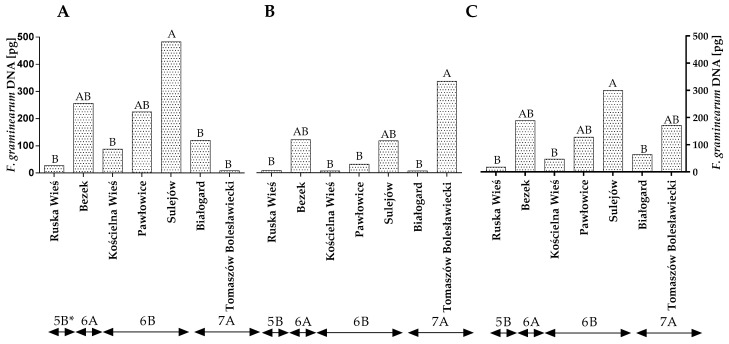
Quantity of *F. graminearum* DNA (pg) in winter wheat grains grown in different locations in Poland (* climatic regions) (**A**) during 2015, (**B**) during 2016 and (**C**) the average over the years of the study. A, B and C—significant at *p ≤* 0.01.

*Fusarium graminearum*, another DON-producing species of the genus *Fusarium*, was more prevalent in winter wheat grains other than *F. culmorum* (Figure 1A–C and Table S2). The amount of *F. graminearum* DNA in the wheat grains was higher in the first year of the study than in the second year (Figure 2A,B). In 2015, the quantity of *F. graminearum* DNA was the highest (224.74 pg) in winter wheat grown in Sulejów and the lowest in winter wheat cultivated in Tomaszów Bolesławicki (8.40 pg) (Figure 2A). In the second year of the study, the results of the qPCR analysis were different. The quantity of *F. graminearum* DNA was the highest in Tomaszów Bolesławicki (334.06 pg) and lowest in Ruska Wieś, Kościelna Wieś and Białogard at 8.14 pg, 6.75 pg and 7.26 pg, respectively (Figure 2B). The qPCR identification of *F. graminearum* revealed differences in the quantity of *F. graminearum* DNA within climate regions 6A (first year of study) and 7A (second year of study) (Figure 2A,B). The average quantity of *F. graminearum* DNA was the highest (299.68 pg) in winter wheat grown in Sulejów and the lowest in winter wheat cultivated in Białogard (63.45 pg), Kościelna Wieś (47.80 pg) and Ruska Wieś (17.80 pg) (Figure 2C). The average quantity of *F. graminearum* DNA was the lowest in the grains of winter wheat grown in region 5B (17.81 pg).

**Figure 3:**
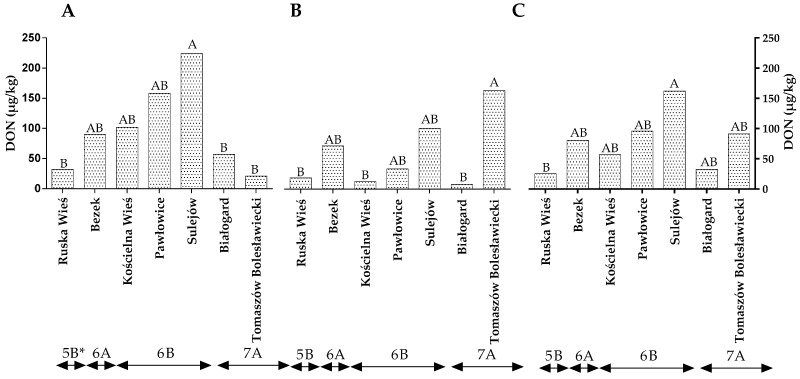
Quantity of DON in winter wheat grains grown in different locations in Poland (* climatic regions) (**A**) during 2015, (**B**) during 2016 and (**C**) the average over the years of the study. a, b and c—significant at *p ≤* 0.05; A, B and C—significant at *p ≤* 0.01.

The level of DON in winter wheat grains did not differ between years of the study (Table S2). The statistical analysis revealed no significant differences in the DON levels between locations representing different climatic regions (Figure 3A–C). In the first year of the study, the level of DON contamination was the highest in Sulejów (224.35 µg/g) and lowest in Ruska Wieś, Białogard and Tomaszów Bolesławicki at 32.47 µg/g, 57.15 µg/g and 21.18 µg/g, respectively (Figure 3A). In the second year of the study, the amount of DON was the highest in the grain from Sulejów and lowest in the grains from Ruska Wieś, Kościelna Wieś and Białogard (Figure 3B). The average DON content of the wheat grains was lowest in Ruska Wieś belonging to region 5B (Figure 3C).

### 2.3. DNA Quantification by qPCR in Other Fungal Species

**Figure 4:**
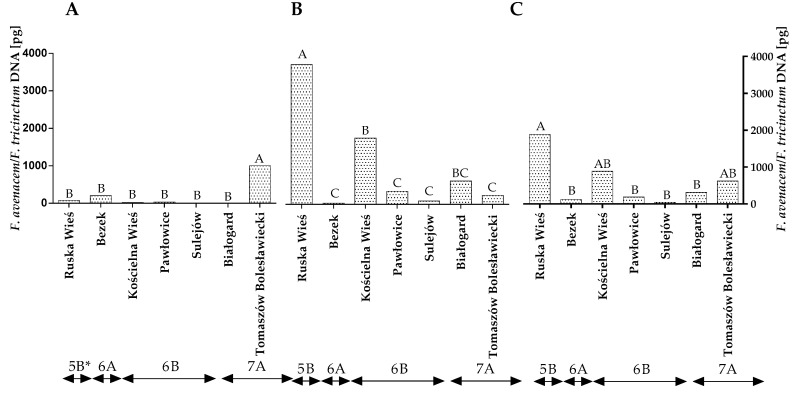
Quantity of *F. avenaceum/F. tricinctum* DNA (pg) in winter wheat grains grown in different locations in Poland (* climatic regions) (**A**) during 2015, (**B**) during 2016 and (**C**) the average over the years of the study. A, B and C—significant at *p ≤* 0.01.

A conventional mycological analysis revealed that winter wheat grains were colonized mainly by *F. avenaceum, F. poae* and *Penicillium* spp. These fungi synthesize various mycotoxins, and they can compete with DON producers. Therefore, a qPCR analysis was carried out to reliably evaluate the contamination of winter wheat grains caused by the above fungi. The statistical analysis demonstrated that the quantity of the DNA of the *F. avenaceum/F. tricinctum* genotype varied significantly across the years of the study, locations and climatic regions (Figure 4A–C and Table S2). The quantity of *F. avenaceum/F. tricinctum* DNA was the highest in Ruska Wieś located in region 5B (1885.9 pg), particularly in 2016.

**Figure 5:**
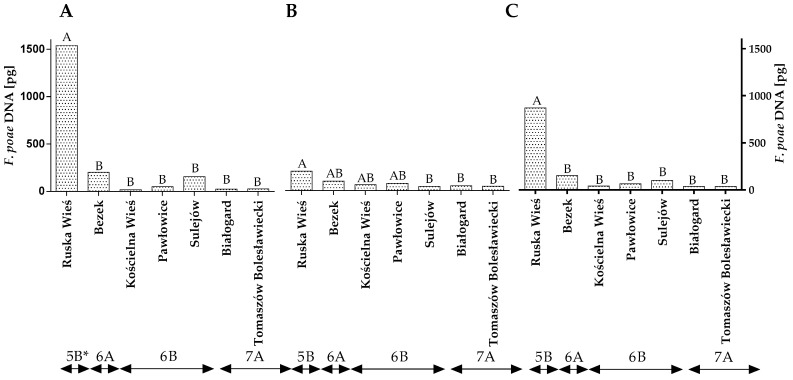
Quantity of *F. poae* DNA (pg) in winter wheat grains grown in different locations in Poland (* climatic regions) (**A**) during 2015, (**B**) during 2016 and (**C**) the average over the years of the study. A, B and C—significant at *p ≤* 0.01.

Similar observations were made in an analysis of *F. poae* DNA. In this case, differences were observed between the years of the study, locations and climatic regions (Figure 5A–C and Table S2). However, no differences were found between locations within climate regions.

The qPCR analysis revealed that, in both years of the study, winter wheat grain grown in Ruska Wieś was more highly contaminated with *F. poae* DNA than grains from the remaining locations (Figure 5A,B). The average quantity of *F. poae* DNA in wheat grain from Ruska Wieś (climatic region 5B) was determined at 871.62 pg (Figure 5B). The analyzed parameter was significantly lower in the remaining locations/climatic regions.

**Figure 6:**
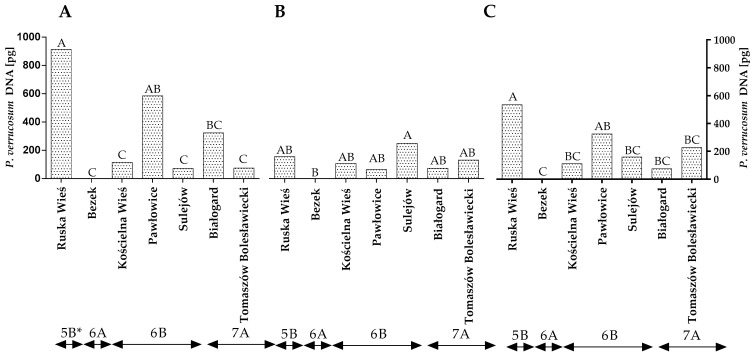
Quantity of *P. verrucosum* DNA (pg) in winter wheat grains grown in different locations in Poland (* climatic regions) (**A**) during 2015 (**B**) during 2016 and (**C**) the average over the years of the study. A, B and C—significant at *p ≤* 0.01.

The DNA of *P. verrucosum*, a major producer of ochratoxin A (OTA), was also quantified by qPCR. The statistical analysis demonstrated that the quantity of *P. verrucosum* DNA varied significantly across the years of the study, locations and climatic regions (Figure 6A–C and Table S2). Differences were also found between locations within the climate regions but only in the first year of the study (Figure 6A). The quantity of *P. verrucosum* DNA was highest in the grain samples from Ruska Wieś in the first year of the study (Figure 6A), whereas only trace amounts of *P. verrucosum* DNA were detected in the grain samples from Bezek (both years of the study) (Figure 6A,B). The statistical analysis confirmed that the climatic conditions of Ruska Wieś (region 5B) favored the development of *P. verrucosum*, whereas the average quantity of the DNA of this fungal species was significantly lower (*p* < 0.01) in the remaining locations/climatic regions (Figure 6C).

### 2.4. The Influence of Climate on the Prevalence of Fungi

Spearman’s rank correlation analysis revealed a significant positive correlation between the ambient temperature in the first ten days of May and the presence of the DNA of the *F. avenaceum/F. tricinctum* genotype in winter wheat grains grown in Poland (R = 0.54; *p* ≤ 0.05) (Table 2). The qPCR assay demonstrated that the *F. avenaceum/F. tricinctum* genotype was most prevalent in grains sampled from Ruska Wieś in 2016, when the mean daily temperature in the first ten days of May (14.3 °C) was higher than in the remaining locations (Table S3). In the remaining locations, the thermal conditions were less conducive to the spread of spike infections (temperatures of 10.9–13.5 °C).

The statistical analysis also revealed that the severity of *F. avenaceum/F. tricinctum* infection in winter wheat grains was significantly affected by the humidity between 11 and 20 May (R = 0.50) (Table 3). In Ruska Wieś, the precipitation reached 42 mm between 11 and 20 May, and it could have contributed to the spread of FHB (Table S4).

The correlation analysis demonstrated that weather conditions had no significant effect on the prevalence of the remaining fungal species in winter wheat grains. However, the statistical analysis revealed certain noteworthy trends. High temperatures between 11 and 20 May promoted the occurrence of *F. graminearum* on wheat grains, which suggests that warm weather contributes to wheat infections caused by this fungal species. A detailed analysis of the effect of temperature on the prevalence of *F. graminearum* showed that, in the first year of the study, the quantity of *F. graminearum* DNA in wheat grains was highest in Sulejów (482.74 pg), where the average temperature between 11 and 20 May was 12.5 °C. The amount of the pathogen’s DNA was somewhat lower in the grain samples from Bezek (256.42 pg) and Pawłowice (224.74 pg), where temperatures reached 13.0 °C and 12.6 °C, respectively. In the second year of the study, the quantity of *F. graminearum* DNA was highest in the grain of wheat grown in Tomaszów Bolesławicki (334.06 pg), where the average temperature between 11 and 20 May was 12 °C. A mathematical analysis revealed no correlation between the quantity of *F. graminearum* DNA in the wheat grains and ten-day precipitation totals. A moderate positive correlation was observed between the ambient temperatures in the first twenty days of July and the content of *F. graminearum* DNA in the grains (Table 2). A detailed analysis revealed that, in both years of the study, air temperature of 19.8 °C promoted the spread of FHB infections (Table S3). Another interesting trend was also noted in July, when a minor negative correlation was observed between the temperature and the quantity of the DNA of the *F. avenaceum/F. tricinctum* genotype, which could suggest that *F. avenaceum* and *F. graminearum* have different temperature requirements or enter into competitive interactions. A Spearman’s rank correlation analysis showed no effect of weather conditions on grain contamination with DON (Table 2 and Table 3).

### 2.5. Correlation between the Number of Fungal Isolates Identified by the Cultural Method and the Quantity of the DNA of Fungal Species

The presence of correlations between the number of fungal isolates obtained from winter wheat grain and the quantity of the DNA of the analyzed fungal species/genotypes was also determined in the statistical analysis (Table 4). The analysis revealed a significant correlation between the number of *F. avenaceum* cultures and the quantity of the DNA of the *F. avenaceum/F. tricinctum* genotype measured in the qPCR assay (R = 0.49, *p* ≤ 0.05).

A higher number *F. avenaceum* cultures tended to be negatively correlated with the quantity of the DNA of DON-producing *F. culmorum* and *F. graminearum* (R = −0.32 and R = −0.30, respectively). An analysis of the remaining *Fusarium* species did not reveal significant correlations due to a smaller number of fungal isolates identified by the culture-based method.

The correlations between the quantity of the DNA of the analyzed fungal species/genotypes and DON levels in the winter wheat grains were also determined in the statistical analysis (Table 5).

A mathematical analysis revealed a negative correlation between the quantity of *F. avenaceum*/*F. tricinctum* DNA and DON levels in winter wheat grains (R = −0.58; *p ≤* 0.05). The deoxynivalenol levels were also significantly correlated with the quantity of the DNA of the DON-producing fungi *F. culmorum* and *F. graminearum* (Table 5). A Spearman’s rank correlation analysis demonstrated significant correlations between the quantity of the DNA of the *F. avenaceum*/*F. tricinctum* genotype and the DNA of *F. culmorum* and *F. graminearum* (R = −0.49 and R = −0.59, respectively; *p ≤* 0.05) (Table 5).

## 3. Discussion

The presence of mycotoxins in food products is not only a serious economic problem, but it also poses a health risk for humans and animals around the world. The occurrence of mycotoxins in agricultural crops is influenced by the local climate [32,33]. Extreme temperatures, drought, high humidity and some agricultural practices can contribute to food and feed contamination with mycotoxins [34]. Inadequate grain storage can lead to the accumulation of mycotoxins, which are responsible for up to 50% of cereal production losses worldwide [6]. FHB reduces the quantity and quality of wheat yields through the selective loss of albumin, gluten proteins and starch in grains [35]. In recent years, the geographic distribution of various toxin-producing fungal species has changed due to extreme weather events [6]. High temperatures, high relative humidity (>70%) and/or high precipitation during the heading and flowering stages contribute to fungal epidemics in wheat stands [36].

In the present study, the grains of the analyzed winter wheat varieties were colonized mainly by *F. avenaceum* and *F. poae*, as well as by *F. culmorum*, *F. equiseti*, *F. graminearum*, *F. oxysporum*, *F. sambucinum*, *F. solani*, *F. sporotrichioides* and *F. tricinctum*. According to the research, the predominant causal agents of FHB can change over time [37], but *F. graminearum* and *F. culmorum* are considered to be the most common [6,27,38].

The results of the mycological analysis and qPCR confirmed that the wheat grain was weakly colonized by *F. culmorum*. The analyzed grain was most abundantly colonized by *F. avenaceum* and *F. poae*, followed by *F. graminearum. Penicillium verrucosum* DNA was also identified in the wheat grain, in particular in cold climatic region 5B, which is consistent with the literature, because this species typically occurs in cold regions [6].

Pathogenic fungal species produce mycotoxins that, under favorable weather conditions, can be accumulated in grains directly after infection. This group of compounds includes DON, which is one of the most frequently identified mycotoxins in crops and plant-based foods in Poland [39]. In this study, DON was detected in all the samples of winter wheat grains within a concentration range of 4.00–311 μg/kg. In the literature, DON has been identified in winter wheat grains at a concentration of 127.95 μg/kg [37]. A four-year study demonstrated that the DON levels in wheat grains cultivated in Poland were generally low and did not exceed 1250 μg/kg, which suggests that the produced grain was safe for human and animal consumption [1]. In another study, DON was the most frequently identified mycotoxin in samples of wheat grains [40]. The cited authors detected DON in 92% of the grain samples in 2017 (5.2–1670.7 μg/kg) and in 61% of the samples in 2018 (95.0–461.7 μg/kg). According to the research, DON is a ubiquitous toxin in the temperate climate [41], as well as in other climatic zones [42,43,44,45]. In a study investigating wheat grain grown in Tunisia, 83% of the analyzed samples were contaminated with DON [42]. This toxin was also detected in other cereals cultivated in Africa, Asia and America [43]. A study conducted in Uruguay in 1993–1995 revealed the presence of DON in wheat grain and other crops [44]. High concentrations of DON in wheat were also reported in Japan [45].

In the present study, the DON levels in the grain samples differed across the examined locations and climatic regions. On average, the DON concentration was the highest in the wheat grain grown in Sulejów [162 μg/kg] and the lowest in the samples obtained from Ruska Wieś [25.4 μg/kg]. Mathematical analyses confirmed that grain contamination with DON was directly associated with the severity of *F. graminearum* and *F. culmorum* infections. The quantity of *F. culmorum* and *F. graminearum* DNA was correlated with the DON levels in the examined grain samples, as demonstrated by the calculated correlation coefficients (R = 0.51 at *p* = 0.05 and R = 0.89 at *p* = 0.01, respectively). The concentration of DON at the site of infection may be reduced as a result of transport by xylem to other plant tissues [46]. Moreover, trichothecenes undergo glycosylation, and mycotoxins can be masked in conventional chemical analyses [47]. Even high levels of grain contamination with mycotoxins are not always directly correlated with the severity of the FHB symptoms [6].

The presence of a correlation between the DON levels and the concentration of the DNA of trichothecene-producing fungi was previously confirmed in a study of qPCR detection of toxin-producing fungi in pig feed [11]. In the current study, an analysis of the correlations between DON levels and the concentrations of *F. avenaceum/F. tricinctum* DNA demonstrated that DON-producing fungi enter into competitive interactions with the *F. avenaceum/F. tricinctum* genotype (R = −0.58 at *p* = 0.05). Moreover, the Spearman’s rank correlation coefficients point to the presence of negative correlations between the quantity of the DNA of *F. culmorum* and *F. graminearum* vs. the *F. avenaceum*/*F. tricinctum* genotype (R = −0.49 and R = −0.59, respectively; *p* = 0.05). Weak negative correlations were also noted between the quantity of *F. graminearum* and *F. culmorum* DNA in the grain and the number of *F. avenaceum* cultures, which could be indicative of competitive interactions in spikes that had been previously infected with *F. avenaceum*. *Fusarium* species that cause FHB to enter into synergistic or competitive interactions. A population study examining the fungal complex causing FHB revealed positive correlations between *F. avenaceum* and *F. culmorum* coinfecting wheat spikes and the absence of such correlations between *F. avenaceum* and *F. graminearum* [48]. However, according to the cited authors, these observations could be attributed to fungal responses to environmental conditions rather than synergy effects. The presence of competitive interactions between *Fusarium* species was postulated in earlier studies, but further research focusing on specific toxin-producing strains is needed to confirm these findings [49]. The isolates of *F. graminearum* and *F. avenaceum* have different mycotoxin profiles. *Fusarium graminearum* produces trichothecenes, whereas *F. avenaceum* synthesizes enniatins (ENNs) [50]. According to the latest research, the toxins produced by *F*. *avenaceum* and *F*. *graminearum* can coinfect wheat plants, but their effects differ [51]. The synergistic activity of DON and ENNs compromises seed germination and plant growth and leads to chlorophyll degradation, but these mycotoxins enter into antagonistic interactions in cell death and the induction of oxidative stress, where DON counteracts the cellular stress produced by ENB. An in vitro study also revealed that ENNs inhibited *F. graminearum* development, whereas DON promoted the growth of *F. avenaceum*, which suggests that ENNs participate in competitive interactions between fungi. This hypothesis was confirmed by an earlier study that demonstrated that the accumulation of ENNs was unrelated to the severity of the disease symptoms in potatoes, peas and durum wheat (FHB) [52].

DON and ENNs could play different roles in the infection of cereal crops by *Fusarium* fungi that synthesize these mycotoxins. DON participates in the infection process and is directly responsible for the symptoms of necrosis and, whereas ENNs do not exert such effects and can be responsible for the effective colonization of plant tissues. However, high concentrations of *F. avenaceum* DNA could be attributed to weather conditions that promote infection. In the present study, in wheat grain samples from Ruska Wieś, *F. avenaceum* DNA was detected mainly in 2016, which was characterized by the highest mean daily temperature recorded in Poland in the first ten days of May (which could be considered anomaly), as well as a relative high humidity between 11 and 20 May. According to the literature, growing greenhouse gas emissions lead to changes in the regional climate patterns and cause seasonal variations that are often extreme. The steady increase in the mean daily temperatures is accompanied by sudden and highly abundant precipitation but also catastrophic drought events [18]. The causal agents of FHB, i.e., *F. avenaceum*, *F. culmorum*, *F. graminearum* and *F. poae*, have different temperature preferences, and selected species can become locally dominant during extreme weather events. These effects can be exacerbated by different toxicogenic potentials of fungal species and strains, which are not always synergistic.

*Fusarium avenaceum* is a major pathogen of crops that causes significant economic losses around the world [53]. Spike infections with *F. avenaceum* can decrease grain yields by up to 25% [54]. This cosmopolitan fungal species can be established as a saprotroph in the soil environment, but it can also infect plants both as a weak and a dominant pathogen [55]. *Fusarium avenaceum* has a wide range of host plant species, and it has been identified as the causal agent of crop infections in the USA, Northern and Central Europe, Australia, South Africa and the arctic and subarctic, as well as in the cold regions of Finland, Norway, Siberia and Canada [53,56].

In many cases, the weather conditions determine the progression and spread of FHB [57]. Excessive precipitation, high humidity and high temperatures during wheat flowering and in the early stages of grain development (watery ripe/early milk) contribute to the development and spread of *Fusarium* infections [58]. In the current study, the quantity of the DNA of the *F. avenaceum/F. tricinctum* genotype was positively correlated with the mean daily temperature in the first ten days of May (R = 0.54, *p* = 0.05) and with the total precipitation between 11 and 20 July (R = 0.50, *p* = 0.05), which confirmed that weather conditions influence grain colonization by the causal agents of FHB.

The severity of disease symptoms and toxin accumulation can vary across different stages of plant growth [36]. Spikes become infected during flowering, when ascospores are released from perithecia and transported by wind and raindrops [59]. Weeds and other crops can also act as a source of infection [60,61]. Infections that occur in the earlier stages of wheat growth are unlikely to be associated with FHB, although *Fusarium* fungi also cause necrosis and dry rot of the basal stem and crown tissue, also known as *Fusarium* crown rot (FCR), of wheat [5]. According to the literature, *F. graminearum* can also infect wheat nodes, but such observations have been rarely made and only in highly susceptible varieties and/or during wet weather [62]. Some authors have argued that infected seedlings do not play a role in the progression of FHB [6], whereas other researchers have concluded that the inoculum from infected seedlings can accumulate on leaves and lead to an outbreak of infection in the flowering stage [61]. In such a case, conidia could also be a source of infection, but they play a less important role in the development of FHB than ascospores, because they are produced under humid and warm conditions and are carried by the wind over much shorter distances [63].

There is considerable evidence to indicate that temperature and humidity are the main environmental factors responsible for the progression of FHB [2,57,64]. The severity of the spike infection caused by *F. avenaceum* increased in response to high precipitation [65]. Other authors found that the concentration of *F. poae* gDNA in wheat grains was influenced by the weather conditions in May and June when cereals develop symptoms of FHB [12]. A positive correlation was also reported between the precipitation levels in May and the concentration of *F. poae* DNA in wheat grains (R = 0.75) [66].

Xu et al. [67] reported that both the severity of FHB symptoms and mycotoxin concentrations in wheat grains increased with the duration of wetness and increasing temperatures. Mycotoxin production was also greatly enhanced by high temperatures (≥20 °C) during the initial infection periods. According to the literature, *F. graminearum* infects spikes at higher temperatures (optimal temperature: 26–28 °C; water activity: 0.88) than *F. culmorum*, which thrives at a temperature of 21 °C and water activity of 0.87 [24]. In another study, the hyphal growth of all strains of *F. graminerum* was significantly inhibited, the metabolism was slowed down and the accumulation of DON decreased at a temperature of 10 °C [68].

In the present study, the weather conditions had no significant effect on the quantity of *F. culmorum* and *F. graminearum* DNA. However, the smaller number of *F. culmorum* cultures and the smaller quantity of *F. culmorum* DNA identified by qPCR in comparison with *F. graminearum* confirmed the trends reported in other studies and indicated that climate change has shifted the geographic distribution of *F. graminearum* to Northern and Central Europe, which were previously dominated by *F. culmorum* [26].

Wenda-Piesik et al. [69] found that a total monthly rainfall of 113.9 mm and a relatively low air temperature in June (monthly average of 15.5 °C) resulted in the highest severity of FHB. In Poland, wheat anthesis extends to June, which is believed to be most critical for FHB development [70]. The findings of the above authors [69] seem to confirm the results of this study, where, in the first year, the mean monthly temperatures in June ranged from 15.0 to 15.6 °C in Tomaszów Bolesławicki and Kościelna Wieś, contributing to very high amounts of *Fusarium* DNA in the wheat grains. The predominant species in the above locations were *F. poae* and *F. avenaceum*. In the second year, the mean monthly temperatures in June were considerably higher (the average temperature in all locations reached 17.6 °C, compared with 15.9 °C in 2015). In 2016, the quantity of *F. avenaceum* DNA increased five-fold due to an increase in temperature, whereas the DNA of *F. poae*, which tends to prefer cooler regions, decreased 3.5 times. The quantity of *F. graminearum* DNA in the wheat grains also varied across the years of the study. In the first year, the quantity *F. graminearum* DNA was the highest in Sulejów, Bezek and Pawłowice, characterized by high average temperatures in June (16.1, 17.5 and 17.1 °C, respectively). In the second year, the quantity *F. graminearum* DNA was the highest in Tomaszów Bolesławicki, where the average temperature in June reached 17.1 °C, while higher temperatures were noted in the remaining locations. It should be stressed that the present findings do not fully explain the relationship between air temperature and wheat grain colonization by *Fusarium* fungi, because individual species can be represented by cold-tolerant strains [68].

Precipitation affects the development of FHB in two ways. High humidity contributes to the onset of infection, and spores are washed off from plant surfaces by rain in its later stages [71]. Abundant but sporadic rainfall can also intensify the symptoms of FHB, because raindrops can carry *Fusarium* spores from the soil inoculum [72]. Precipitation can also reduce the efficacy of fungicides, depending on its frequency after treatment [73]. In another study, the response of the grain yield to fungicide application was significantly influenced by rainfall in May and June [57]. The cited authors concluded that fungicide treatments against FHB are justified when the precipitation levels are high during the flowering and heading stages.

In this study, the DON levels differed in the grain samples collected from various locations/climatic regions. On average, the DON concentration was highest in the wheat grains grown in Sulejów (162 μg/kg) and lowest in the grain samples from Ruska Wieś (25.4 μg/kg) in climatic region 5B. These results are consistent with the findings of other authors who have demonstrated that weather conditions affected the DON levels in wheat grains grown in Poland [40]. In the cited study, the DON concentrations were significantly higher in wheat grains grown in Southern compared to Northern Poland, which was confirmed by this research because climatic region 5B occupies Northeastern Poland. According to the cited authors, the dry and hot summer of 2018 not only decreased the wheat yields but also inhibited the development of *Fusarium* pathogens, which is why the mycotoxin levels were low in the analyzed grains [40]. However, an earlier study conducted by the same researchers produced contrary results, and the mycotoxin levels were highest in the grains grown in Northeastern Poland, where the concentration of DON peaked at 1264.5 μg/kg. The observed differences in the grain contamination levels were attributed mainly to weather conditions [74]. In another experiment, DON was identified in nearly all the grain samples, and the DON concentration was highest (2265 μg/kg) in a year characterized by high precipitation during the growing season [41]. In the present study, weather conditions had no effect on the presence of the DNA of DON-producing fungi or DON itself in the grains, which could be attributed to a relatively low grain contamination with *F. graminearum* and *F. culmorum* and high contamination with *F. poae* and *F. avenaceum*. However, climate change undoubtedly affects the prevalence of fungal species that cause FHB, and further research is needed to evaluate the impact of climate change on the populations of pathogenic fungi and the mycotoxin levels in cereal grains.

## 4. Conclusions

1. *Fusarium avenaceum* and *F. graminearum* were the predominant fungal species in the winter wheat grains cultivated in most of the analyzed locations in Poland.
2. The prevalence of fungal pathogens was influenced by weather conditions during the growing season of winter wheat, and DON-producing species were predominant in climatic regions 6A and 6B, which also contributed to higher grain contamination with DON.
3. *Fusarium avenaceum, F. poae* and *P. verrucosum* were the predominant species in the winter wheat grains grown in the coldest climatic region.
4. Mathematical analyses revealed a positive correlation between the quantity of *F. avenaceum* DNA (pg) and temperatures (°C) in the first ten days of May, as well as humidity (mm) between 11 and 20 May.
5. Spearman’s rank correlation analysis confirmed the presence of competitive interactions between enniatin-producing *F. avenaceum* and DON-producing *F. culmorum* and *F. graminearum*.
6. A positive correlation was observed between the presence of *F. culmorum* and *F. graminearum* DNA and DON contamination of winter wheat grains.

## 5. Materials and Methods

### 5.1. Field Experiment

The study involved field experiments that were carried out between 2014 and 2016 in the Experimental Stations for the Evaluation of Crop Varieties (SDOO) in different climatic regions in Poland, consistent with the European climate zones: SDOO Białogard, SDOO Bezek, SDOO Kościelna Wieś, SDOO Ruska Wieś, SDOO Sulejów, SDOO Tomaszów Bolesławiecki and SDOO Pawłowice (Table 6 and Figure S1).

The grains of winter wheat var. Artist and Kilimanjaro (characterized by different winter hardiness and resistance to FHB; Artist—moderate resistance; RGT Kilimanjaro—relatively high resistance) were randomly sampled. Ten grain samples collected from each analyzed location were pooled into composite samples of 1 kg. Wheat grain samples were taken in accordance with ISO 24333 [75]. The samples were analyzed in the laboratory of the Department of Entomology, Phytopathology and Molecular Diagnostics of the University of Warmia and Mazury in Olsztyn.

### 5.2. Isolation and Identification of Fungi by the Culture-Based Method

The taxonomic composition of fungal communities colonizing wheat grains was determined in the first stage of the study. One hundred kernels were selected randomly from each sample and were rinsed with distilled water, rinsed with 70% ethanol for 5 min and with 1% sodium hypochlorite (NaOCl) solution for 5 min and rinsed three times with sterile distilled water. The prepared specimens were placed on PDA in Petri dishes (7 kernels per dish) and incubated at a temperature of 20–23 °C for 7–10 days under laboratory conditions. Next, fragments of the emerged mycelia were transferred onto fresh PDA in sterile Petri dishes. After 14–20 days, the emerged fungal colonies were examined under an optical microscope and identified to the genus and species levels based on morphological traits, taxonomic keys and monographs [55,76,77].

### 5.3. Extraction and Quantification of Genomic DNA from *Fusarium* fungi Colonizing Winter Wheat Grain

DNA was isolated from winter wheat grains with the Maxwell 16 instrument and Maxwell 16 DNA Purification Kit (Promega, Madison, WI, USA). Randomly selected grain samples of 20 g each (in three biological replicates) were ground in the IKA A11 Basic Analytical mill (IKA-Werke, Staufen, Germany) and then in a mortar filled with liquid nitrogen for 45 s. Ground grains were transferred to 1.5-mL Eppendorf tubes, and CTAB extraction buffer and RNase A (Promega) were added. The samples were incubated for 30 min in a thermomixer (Eppendorf, Hamburg, Germany). DNA was extracted with the Maxwell 16 instrument according to the manufacturer’s instructions. The purity and quantity of the isolated DNA were determined with the NanoDrop 2000C spectrophotometer (Thermo Scientific, Waltham, MA, USA). The extracted DNA was stored at a temperature of 4 °C until further analysis.

### 5.4. Identification of Selected *Fusarium* Species and P. verrucosum by qPCR

A quantitative qPCR method was used to assess the presence of DNA from the selected fungal species. The isolated gDNA was used as the matrix in real-time PCR. qPCR was conducted in the ABI 7500 FAST system (Applied Biosystems, Waltham, MA, USA) with the use of Applied Biosystems reagents, specific primers and probes (Table 7) based on the protocols for the analyzed fungal species. The reaction mix of 25 µL contained: 12.5 µL of the TaqMan Universal PCR Master Mix (Applied Biosystems, USA), 10 pM of each primer, 10 pM of each primer labeled with FAM at the 5′ and 6′ ends and TAMRA quencher at the 3′ end, 4.5 µL of deionized water and 5 µL of DNA. The amplification conditions were as follows: initial denaturation at 95 °C for 5 min, followed by 40 cycles of denaturation at 95 °C for 15 s, primer annealing at 60 °C for 15 s and strand synthesis at 72 °C for 1 min. The quantity of DNA isolated from each fungal species and genotype was calculated with the use of calibration curves (Table 7) and the methods described by Livak and Schmittgen [78] and Pfaffl [79] with some modifications.

### 5.5. Detection and Quantification of DON

Samples of ground grains of 25 g each (in three replicates) were placed in 250-mL conical flasks. The samples were extracted in 200 mL of deionized water by shaking on a mechanical shaker for 1 h. The extracts were filtered and purified on an IAC column. The samples were transferred to affinity columns with the DONtest WB kit (VICAM, Milford, MA, USA). The extract was passed through affinity columns, and the columns were washed with 5 mL of deionized water. The eluate was collected into a glass tube, and the solvent was evaporated in a stream of nitrogen. The residue was dissolved in the mobile phase (0.5 mL) for the HPLC assay, and it was shaken on a mechanical shaker. The extracts were subjected to a chromatographic analysis in the Shimadzu LC-20AD system (Tokyo, Japan) under the following conditions: chromatographic column—Jupiter 5u C18 300A (Phenomenex, Torrance, CA, USA), 250 × 4.60 mm; column temperature—24 °C; mobile phase—water:acetonitrile (900 mL:100 mL); flow rate—1 mL/min; injection volume—100 µL and UV detector, wavelength—220 nm. The concentrations of DON were determined by correlating the peak areas of the samples with the standard curve obtained by HPLC analysis of the standard solution. The limit of quantification of the method used was LOQ = 10 μg/kg, the repeatability expressed as the coefficient of variation was <10% and the accuracy expressed as recovery exceeded 93%.

### 5.6. Statistical Analysis

Data were processed statistically in Dell Statistica v. 13 (software.dell.com, accessed on 31 November 2021). The significance of the differences between the average gDNA yield of each fungal species was evaluated with Tukey’s HSD test at *p* = 0.01. The strength of the associations between the examined variables was determined by calculating the values of the Spearman’s rank correlation coefficients (*R*).

## Figures and Tables

**Table 1:** The effects of the main factors (location and winter wheat variety) and their interactions on the quantity of DNA of the selected fungi and DON levels in winter wheat grains determined by two-way ANOVA.

Parameter	Location (L)	Variety (V)	L × V
*F. avenaceum/F. tricinctum DNA*	**	ns	**
*F. culmorum DNA*	*	ns	*
*F. graminearum DNA*	**	ns	**
*F. poae DNA*	***	*	***
*P. verrucosum DNA*	***	ns	***
DON (µg/kg)	**	ns	*

* Significant at *p* ≤ 0.05, ** significant at *p* ≤ 0.01, *** significant at *p* ≤ 0.001 and ns—not significant.

**Table 2:** Correlation between temperature (°C) and the quantity of fungal DNA in winter wheat grains (mean values for years of the study and locations).

Month	Days		DNA Quantity				DON (µg/kg)
		*F. avenaceum/F. tricinctum*	*F. culmorum*	*F. graminearum*	*F. poae*	*P. verrucosum*	
May	1–10	0.54 *	−0.20	−0.16	−0.19	−0.23	−0.22
	11–20	0.09	0.18	0.31	−0.22	−0.30	0.18
	21–31	0.35	−0.20	−0.18	−0.20	−0.26	−0.18
June	1–10	0.06	0.19	0.18	−0.09	−0.28	0.16
	11–20	0.03	0.12	0.20	−0.13	−0.25	0.16
	21–30	0.33	−0.15	−0.19	−0.23	−0.30	−0.14
July	1–10	−0.39	0.17	0.37	0.06	−0.01	0.16
	11–20	−0.07	0.21	0.35	−0.30	−0.29	0.26
	21–31	0.25	0.09	0.02	−0.30	−0.25	0.09

* R—significant at *p ≤* 0.05.

**Table 3:** Correlation between precipitation levels (mm) and the quantity of fungal DNA in winter wheat grains.

Month	Days		DNA Quantity				DON (µg/kg)
		*F. avenaceum/F. tricinctum*	*F. culmorum*	*F. graminearum*	*F. poae*	*P. verrucosum*	
May	1–10	−0.02	−0.04	0.09	0.25	0.18	0.08
	11–20	0.50 *	−0.21	−0.23	0.02	−0.10	−0.25
	21–31	−0.07	−0.28	0.15	−0.06	−0.21	0.01
June	1–10	0.09	−0.20	−0.13	−0.24	0.02	−0.06
	11–20	0.06	−0.27	0.17	−0.24	−0.24	0.12
	21–30	−0.22	0.08	−0.22	0.01	0.17	−0.18
July	1–10	0.24	−0.18	−0.22	−0.11	−0.12	−0.27
	11–20	0.12	−0.16	0.06	−0.26	−0.15	0.13
	21–31	−0.02	−0.19	−0.22	0.13	0.02	−0.27

* R—significant at *p ≤* 0.05.

**Table 4:** Correlation between the number of fungal isolates identified by the culture-based method and the DNA quantity determined by qPCR.

DNA Quantity	Number of Isolates				
	*F. avenaceum*	*F. tricinctum*	*F. culmorum*	*F. graminearum*	*P. verrucosum*
*F. avenaceum*/*F. tricinctum*	0.49 *	0.26	0.15	−0.11	−0.19
*F. culmorum*	−0.32	−0.17	0.27	−0.04	0.11
*F. graminearum*	−0.30	0.07	0.11	−0.15	−0.06
*F. poae*	0.00	0.18	−0.25	0.17	−0.22
*P. verrucosum*	0.01	0.02	−0.06	0.18	0.03

* R—significant at *p ≤* 0.05.

**Table 5:** Correlation between the quantity of fungal DNA determined by qPCR and DON levels in winter wheat grains.

DNA Quantity	qPCR Detection (DNA)				DON (µg/kg)
	*F. culmorum*	*F. graminearum*	*F. poae*	*P. verrucosum*	
*F. avenaceum*/*F. tricinctum*	−0.49 *	−0.59 *	−0.04	−0.03	−0.58 *
*F. culmorum*	-	0.32	−0.09	0.13	0.51 *
*F. graminearum*	-	-	−0.11	−0.09	0.89 **
*F. poae*	-	-	-	0.09	−0.14
*P. verrucosum*	-	-	-	-	−0.01

* R—significant at *p ≤* 0.05; ** R—significant at *p ≤* 0.01.

**Table 6:** Climatic regions in Poland [19].

Location	GPS	Climatic Region
Białogard	φ = 54°00′, λ = 16°00′, H = 24 m a.s.l.	7A
Bezek	φ = 51°11′, λ = 23°15′, H = 224 m a.s.l.	6A
Kościelna Wieś	φ = 51°48′, λ = 18°01′, H = 120 m a.s.l.	6B
Ruska Wieś	φ = 53°53′, λ = 22°28′, H = 130 m a.s.l.	5B
Sulejów	φ = 51°35′, λ = 19°86′, H = 188 m a.s.l.	6B
Tomaszów Bolesławiecki	φ = 51°17′, λ = 15°41′, H = 200 m a.s.l.	7A
Pawłowice	φ = 50°28′, λ = 18°29′, H = 240 m a.s.l.	6B

φ—latitude, λ—longitude and H—meters above sea level.

**Table 7:** qPCR primers and probes used in the identification of *Fusarium* species and *P. verrucosum*.

Genotype/Gene	Primer/Probe	Sequence (5′-3′)	Regression Equation, Efficiency of qPCR (E)	References
*F. avenaceum*/*F. tricinctum*	Avetric f	5′-AGCAGTCGAGTTCGTCAACAGA-3′	y = −3.35x + 37.21	[80]
*Esyn1*	Avetric r	5′-GGCYTTTCCTGCGAACTTG-3′	E = 98.5	
	Avetric probe	FAM—CCGTCGAGTCCTCT—MGB	R^2^ = 0.99	
*F. culmorum*	FculC561 fwd	5′-CACCGTCATTGGTATGTTGTCACT-3′	y = −3.49x + 35.45	[81]
*EF1α*	FculC614 rev	5′-CGGGAGCGTCTGATAGTCG-3′	E = 93.6 R^2^ = 0.98	
*F. graminearum*	FgramB379 fwd	5′-CCATTCCCTGGGCGCT-3′	y = −3.29x + 33.32	[81]
*EF1α*	FgramB411 rev	5′-CCTATTGACAGGTGGTTAGTGACTGG-3′	E = 100 R^2^ = 0.97	
*F. poae*	Poae f	5′-GCGGCCGCTTTTGTCA-3′	y = −3.2x + 33.85	[80]
*Esyn1*	Poae r	5′-GCCTTTCCAGCAAGAGATGGT-3′	E = 99.8	
	Poae probe	FAM—AAAGCGGTCGAGTCTG—MGB	R^2^ = 0.99	
*P. verrucosum*	rRNA forward	5′-TAAGGTGCCGGAATACACGCTCAT-3′	y = −3.53x + 22.27	[82]
rRNA	rRNA reverse	5′-TAGTTCATTCGGCCCGTGAGTTGT-3′	E = 92.7	
	PV rRNA-Probe	Fam-TCTAGACAGCCCGACGGTGGCCATGGAAGT-Tamra	R^2^ = 0.99	

## Data Availability

Not applicable.

## Supplementary Materials

The following are available online at https://www.mdpi.com/article/10.3390/toxins14020102/s1: Table S1. Fungi isolated from winter wheat grains. Table S2. Results of qPCR detection of fungal DNA and DON contamination in wheat grains across the locations and years of the study. Table S3. Mean ten-day air temperatures in 2014–2016 (°C) across locations (* climatic regions). Table S4. Ten-day precipitation totals in 2014–2016 (°C) across locations (* climatic regions). Figure S1. Map of Poland, including specific locations and climatic regions (7B* zone not represented in this study).
