# Supplementary material for: Prevalence of Fusarium fungi and Deoxynivalenol Levels in Winter Wheat Grain in Different Climatic Regions of Poland
Source: Toxins (Basel). 2022 Jan 28;14(2):102. doi: 10.3390/toxins14020102 (PMC8877411; doi:10.3390/toxins14020102)
Supplement: Supplementary file 1 [file toxins-14-00102-s001.zip › toxins-1517227-supplementary.pdf]

Supplementary Materials: Prevalence of *Fusarium* fungi and Deoxynivalenol Levels in Winter Wheat Grain in Different Climatic Regions of Poland

Adam Okorski, Alina Milewska, Agnieszka Pszczółkowska, Krzysztof Karpiesiuk, Wojciech Kozera, Joanna A. Dąbrowska and Justyna Radwińska

Table S1. Fungi isolated from winter wheat grain.

Species/Genus	Bezek		Białogard		Kościelna Wieś		Pawłowice		Ruska Wieś		Sulejów		Tomaszów Bolesławiecki		Total
	2015	2016	2015	2016	2015	2016	2015	2016	2015	2016	2015	2016	2015	2016	
Alternaria alternata	86	134	60	66	52	124	65	102	88	23	43	91	58	123	1115
Aureobasiudium bolleyi				1										2	3
Cladosporium cladosporioides			1				2		1	1	2		2	3	12
Drechslera biseptata			2					4				1			7
Drechslera tritici-repentis		4													4
Epicocum nigrum	29	14	7	9	3	16	79	7	41	3	30	9	11	14	272
Fusarium avenaceum			1	10		3			1	8					23
F. culmorum			3	2					1	2					8
F. equiseti		1													1
F. graminearum		2								1					3
F. oxysporum				1						1		1			3
F. poae	4	3	1	2	1	1	4	3	9	2	6	1	1	2	40
F. tricinctum	2							1	2				5		10
F. sporotrichioides										1		1			2
Fusarium spp.				1	2					2	1	1			7
Bipolaris sorokiniana												1			1
Nigrospora oryzae		2				1									3
Penicilium spp.	1				8		1	3		1		2			16
Rhizopus nigricans				2			4		8	16	4		2	3	39
Stemphylium spp.		2				5						1			8
Trichoderma spp.								9	2						11
Non-sporulating fungi			10												10
Total	122	162	85	94	66	150	155	129	153	61	86	109	79	147	1598

Table S2. Results of qPCR detection of fungal DNA and DON contamination in wheat grain across locations and years of the study.

Location (A)	Year (B)	*F. culmorum*	*F. graminearum*	*F. avenaceum/ F. tricinctum*	*F. poae*	*P. verrucosum*	DON (µg/kg)
Ruska Wieś	2015	1.24	27.47	76.34	1540.53	912.63	32.48
	2016	0.58	8.14	3695.52	202.71	155.02	18.38
Bezek	2015	1.50	256.43	200.22	202.69	1.41	90.30
	2016	1.30	121.09	35.95	98.73	0.44	70.80
Kościelna Wieś	2015	8.85	87.62	20.04	17.54	114.76	102.03
	2016	0.95	6.76	1751.45	60.86	106.86	12.00
Pawłowice	2015	10.17	224.74	34.31	49.92	585.40	158.30
	2016	0.00	31.06	344.06	76.56	64.80	33.08
Sulejów	2015	1.69	482.75	0.00	157.33	72.81	224.35
	2016	2.46	116.63	90.23	43.64	246.96	99.68
Białogard	2015	1.99	119.65	6.99	23.60	75.82	57.15
	2016	0.00	7.26	617.58	50.07	72.72	8.00
Tomaszów Bolesławiecki	2015	0.00	8.40	1000.16	27.90	324.09	21.18
	2016	2.08	334.06	239.72	46.13	130.60	161.48
Ruska Wieś		0,91	17.81	1885.93	871.62	533.82	25.43
Bezek		1,04	132.28	1947.87	202.70	78.21	54.34
Kościelna wise		1,40	188.76	118.08	150.71	0.92	80.55
Pawłowice		5,07	104.35	27.99	58.13	57.60	86.41
Sulejów		4,90	47.19	885.74	39.20	110.81	57.01
Białogard		5,56	115.75	892.88	55.39	346.13	85.15
Tomaszów Bolesławicki		5,08	127.90	189.19	63.24	325.10	95.69
2015		3,63	172.44	191.15	288.50	298.13	97.97
2016		1,05	89.29	967.79	82.67	111.06	57.63
Tukey HSD test (LSD at p-0.01)	AxB	4.68	301.6381	1227.371	807.8753	293.3556	146.26
	A	3.31	213.2903	867.8825	571.2541	207.4338	103.42
	B	1.77	114.0085	463.9027	305.3482	110.8780	42.21

Table S3. Mean ten-day air temperatures in 2014-2016 (°C) across locations (*climatic regions).

Year	Month	Days	Ruska Wieś	Bezek	Kościelna Wieś	Pawłowice	Sulejów	Białogard	Tomaszów Bol.
			5B*	6A		6B			7A
2014	Sep	1-10	15.4	16.8	16.4	17.3	15.7	15.3	15.9
		11-20	16.2	16.5	17.2	17.1	15.8	15.5	16.1
		21-30	11.4	11.6	12.6	12.3	11.3	12.3	12.1
	Oct	1-10	10.6	11.7	12.7	12.6	11.4	12.6	12.9
		11-20	11.2	14.4	13.5	13.9	12.7	12.9	13
		21-31	3.4	4.8	6.4	6.7	4.8	7.9	6.8
	Nov	1-10	6.7	10.5	9.4	9.4	9.6	8.6	8.2
		11-20	3.1	5.1	6.8	8.7	6	6.8	6.6
		21-30	-2.3	-2.2	1.5	2	-0.2	2.6	1.5
	Dec	1-10	-3.2	-3.1	-0.3	-0.7	-1.4	-0.5	-0.1
		11-20	2.6	4.4	4.6	4.5	3.3	4.6	4.8
		21-31	-3.3	-1.6	0	1.2	-1.3	0.9	1.6
2015	Jan	1-10	-2.1	-2.2	1	-1.7	-0.7	2.6	2.4
		11-20	1.3	3.4	3.5	4.3	3	3.9	3.3
		21-31	-1.3	0.9	0.7	0.4	-0.6	0.8	0.5
	Feb	1-10	-2.4	-1.9	-0.8	-3.7	-2.6	0	-1.2
		11-20	-1.5	0.2	1.8	0.4	0.5	1.5	0.9
		21-28	2.1	5.2	4.6	3.9	3.3	3.1	2.3
	Mar	1-10	3.4	3.8	4.6	3.4	3.4	4.6	4.4

		11-20	4.7	4.7	5.3	4.8	3.7	4.3	4
		21-31	5.3	7.6	5.9	7.8	5.6	4.8	5.3
	**Apr**	1-10	4.6	4.5	4.4	4.1	3.7	4.7	3.8
		11-20	7.6	8.5	9.1	9.6	7.5	8.2	8.3
		21-30	10.9	12.5	11.5	11.5	10.7	9.6	9.9
	**May**	1-10	11.2	13.1	12.1	12.7	12.1	10.9	11.9
		11-20	11.1	13	13	12.6	12.5	10.8	11.3
		21-31	12.6	13.9	13.7	12.2	12.7	10.9	12.1
	**Jun**	1-10	15.5	18.8	17.5	18.8	16.9	13.3	15.2
		11-20	15.3	17.2	16.7	17.1	16.2	13.8	15.2
		21-30	14.7	16.6	15.7	15.4	15.4	14.2	14.6
	**Jul**	1-10	19.4	21.2	20.9	21	20.1	19.8	19.8
		11-20	16.6	19.8	20.4	21.9	19.8	16.4	19.3
		21-31	17.1	20.6	19.8	21.8	19.7	16.3	19
	**Aug**	1-10	22.3	24.5	24.2	23.8	23.7	20.1	23.8
		11-20	20.2	21.5	23.3	23.7	22.5	20.7	22.7
		21-31	19.3	21.3	20.6	20.9	20.1	18.7	19.2
	**Sep**	1-10	14.8	16.2	15.9	15.9	15.7	14.5	15.1
		11-20	16.3	18	17.6	18	17.9	15.4	16.1
		21-30	12.9	13.3	12.8	12.2	12.4	11.4	10.6
	**Oct**	1-10	7.9	9.4	9.9	9.7	9.1	9.1	9.4
		11-20	6	6.8	5.8	7.2	6.6	5.9	4.4
		21-31	5.6	6.6	7.5	8.1	7.3	7.6	7.4
	**Nov**	1-10	5.5	5.9	7.6	7.6	6.3	8	7.9
		11-20	7.2	8.8	9.1	9.3	9	8.8	9.3
		21-30	0	1.3	1.7	0.6	1.1	2.6	1.9
	**Dec**	1-10	4.3	4.2	6.6	4.8	5.6	6.1	6.1
		11-20	2.8	3	4.2	3.9	4	4.5	5.9
		21-31	1.8	3.5	5.6	4.1	4.3	5.9	6.4
**2016**	**Jan**	1-10	-11	-8.3	-6.5	-8.1	-7.3	-7.6	-4
		11-20	-5.2	-3.9	-2.2	-1.9	-2.7	-0.7	-3.5
		21-30	-1.2	-1.1	1.6	0.9	0.1	2.1	1.6
	**Feb**	1-10	3.1	4.3	6.5	5.2	5.2	5.5	5.1
		11-20	0.8	3.6	3.2	3	2.8	0.7	2.1
		21-28	0.5	2.7	3.9	3	2.7	2.2	2.4
	**Mar**	1-10	1.8	4	2.9	3.4	3.4	2.2	1.2
		11-20	1.3	3.3	2.8	2.8	2.7	3	2.6
		21-31	4.8	4.6	6.3	6.6	5.5	5.2	6.2
	**Apr**	1-10	8.9	10.7	10.1	9.3	9.6	9.1	8.8
		11-20	9.3	10.4	9.9	9.2	10.1	8.8	8.3
		21-30	6.1	7.3	7.1	5.9	6.6	5.8	5.6
	**May**	1-10	14.3	13.5	13.5	12.5	12.9	12.6	11
		11-20	11.9	12	13.4	11.7	11.7	11.7	12
		21-31	17.9	18.2	19.4	17.4	18.3	19	16.6
	**Jun**	1-10	16.2	16.2	17.7	16.3	15.8	16.8	16.8
		11-20	16	17.3	17.3	16.8	17.1	15.1	16
		21-30	20.4	21.6	21.2	20.9	21.2	19.7	18.7
	**Jul**	1-10	17.4	18.9	18.7	17.8	18	17.5	17.3

		11-20	17.9	19.3	18.9	18.3	18.3	17.5	17.6
		21-31	20.5	22	21.3	20.3	20	18.9	19.9
	Aug	1-10	18	20.2	18.7	19.2	18.8	16.9	17.2
		11-20	15.5	17.1	16.9	16.6	16.3	15.6	15.8
		21-31	19.1	20	19.2	18.5	18.6	18.3	18.2

Table S4. Ten-day precipitation totals in 2014-2016 (°C) across locations (*climatic regions).

Year	Month	Days	Ruska Wieś	Bezek	Kościelna Wieś	Pawłowice	Sulejów	Białogard	Tomaszów Bol.
			5B*	6A		6B		7A	
2014	Sep	1-10	3.4	18.7	49.6	54.5	11.7	21.1	35.4
		11-20	1.6	5.2	17.8	31.5	12.2	32.2	20.4
		21-30	18.1	21.5	9.2	26	16.1	36.1	11.1
	Oct	1-10	0	0	0.4	0.2	13.9	3.9	0
		11-20	23.4	2.5	8.3	12.7	11.2	33.9	14.5
		21-31	0	27.8	13.6	40.6	13.6	3.6	22.7
	Nov	1-10	9.4	0.8	6	13.3	19.2	2.4	3.3
		11-20	0.6	8.4	8	9.7	3.5	5	8.9
		21-30	4.6	5.9	5.7	0	6.7	6.8	3
	Dec	1-10	1	0.8	0	3.2	0.5	3.9	0.3
		11-20	16	12.5	15.3	9.1	23	42.9	26.8
		21-31	15.5	27.6	10.6	12	20.3	91.1	9.3
2015	Jan	1-10	25.4	12.3	13.7	26.9	13.6	36.2	33.8
		11-20	13.4	4.2	1.9	12	8.2	12.4	8
		21-31	22.6	15.8	6.1	17	14.2	11.7	14.6
	Feb	1-10	11.1	2.3	7.8	9.3	6.8	8.7	4
		11-20	1.2	0.2	0.1	0.5	0.2	0	0.1
		21-28	3.6	0.7	0.4	6.6	9.8	2	0.1
	Mar	1-10	12.8	4.9	2	5.5	10.2	21.3	8.8
		11-20	0	14.2	5.2	10.9	8.6	0	9.7
		21-31	15.6	19.5	21.1	13.7	28.5	44.1	35.3
	Apr	1-10	12	11.2	12.7	6.7	8.8	6.8	6.6
		11-20	2.4	5.8	0	0.7	2.2	5.1	2.1
		21-30	6	16.7	4.1	3.4	29.6	13.4	11.8
	May	1-10	71.5	15.2	9.1	20.5	28.5	32.3	17.2
		11-20	18.8	3.7	8.1	5.4	21.2	25.1	11.4
		21-31	9.5	43.3	0	15.1	16.8	7.3	2.1
	Jun	1-10	0.4	2.4	2	2.4	6.5	1	20.2
		11-20	1	6.7	5.2	24.3	35.7	50	25.2
		21-30	21.1	6.4	19.8	21.7	8.2	44.2	19.9
	Jul	1-10	12.2	16.2	8.8	4.6	6.4	11.7	19.5
		11-20	9.2	14.3	13.3	29.9	44.2	18.4	42.7
		21-31	38.4	14.9	7.8	9.8	18.4	54.6	11.8
	Aug	1-10	1	0	0	3.5	2	5.1	1.4
		11-20	0	0	10.2	7	16.7	0	6.7
		21-31	24	6.9	3.4	5.4	3.4	12.6	4.4
	Sep	1-10	19.1	36.5	7	26.1	15.5	64.5	29.9
		11-20	55.4	24.4	8.7	12.2	18.1	20.5	4.6

		21-30	5.2	33.4	0	0.5	2.4	9.9	0
	**Oct**	1-10	0	0	0	0	0	0	4.9
		11-20	7.6	23.9	36.8	18.4	40.7	23.9	46.7
		21-31	7.6	20.4	1.9	3.1	1	1.7	0.7
	**Nov**	1-10	56.8	10.5	6.8	8.3	5.8	29.2	6.5
		11-20	49	27.9	23.2	52.1	26	39.9	37
		21-30	3.2	8.3	9.7	17.9	11.1	19.9	16.5
	**Dec**	1-10	4	3.3	3.5	8	1.3	5.4	4.4
		11-20	28.2	18.7	14.4	11.4	13.8	32.7	11.3
		21-31	28.2	2	1.7	2.5	2.5	36.7	0.5
**2016**	**Jan**	1-10	1.3	7.5	5.8	2.3	6.4	6.5	7.9
		11-20	11.5	23.4	15.1	28.5	23	7.5	18.6
		21-30	14	11.1	9	14.3	7.1	26.5	22.6
	**Feb**	1-10	28.2	3.9	8	26	28	12.6	11.1
		11-20	29.6	28.6	20.6	40.8	29.2	20.8	9.4
		21-28	19.2	22.4	21.4	25.1	26	18.6	27.9
	**Mar**	1-10	11.2	30.7	15.3	13.1	13.5	10.2	18.4
		11-20	7	8.5	5.9	7.6	6.8	0.5	10.7
		21-31	8.2	12.4	21.8	4.9	18.9	15	27.1
	**Apr**	1-10	1.4	15.4	18.7	28.7	18.7	0.6	23.1
		11-20	7.4	28	13.9	16.4	8.3	14.6	13.1
		21-30	22.4	20.2	6.1	11.9	11.7	12.9	6.3
	**May**	1-10	0	6	1.5	24.6	6.9	0	15.3
		11-20	42	43	11.2	7	12.9	35.2	1.5
		21-31	7.6	1.5	21	14.2	21.7	38.7	16.8
	**Jun**	1-10	5.8	1.6	10.4	11.9	26.5	10.7	6.9
		11-20	31	56.4	51.6	20.3	21.8	42.1	80.8
		21-30	10.2	1.8	3.3	29.1	28.3	30	7.9
	**Jul**	1-10	39.4	14.2	8.9	31.1	23.9	83.9	28.8
		11-20	62.3	56.2	60.9	57.7	136.6	51.5	95
		21-31	31.9	61.7	26	22	25.6	36.3	8.8
	**Aug**	1-10	38.6	27.9	5.8	29.4	24.1	37.2	27.3
		11-20	13	5.4	0.9	0.5	0.1	36	23.8
		21-31	23.2	17.2	25.5	11.6	11	6.4	10.7

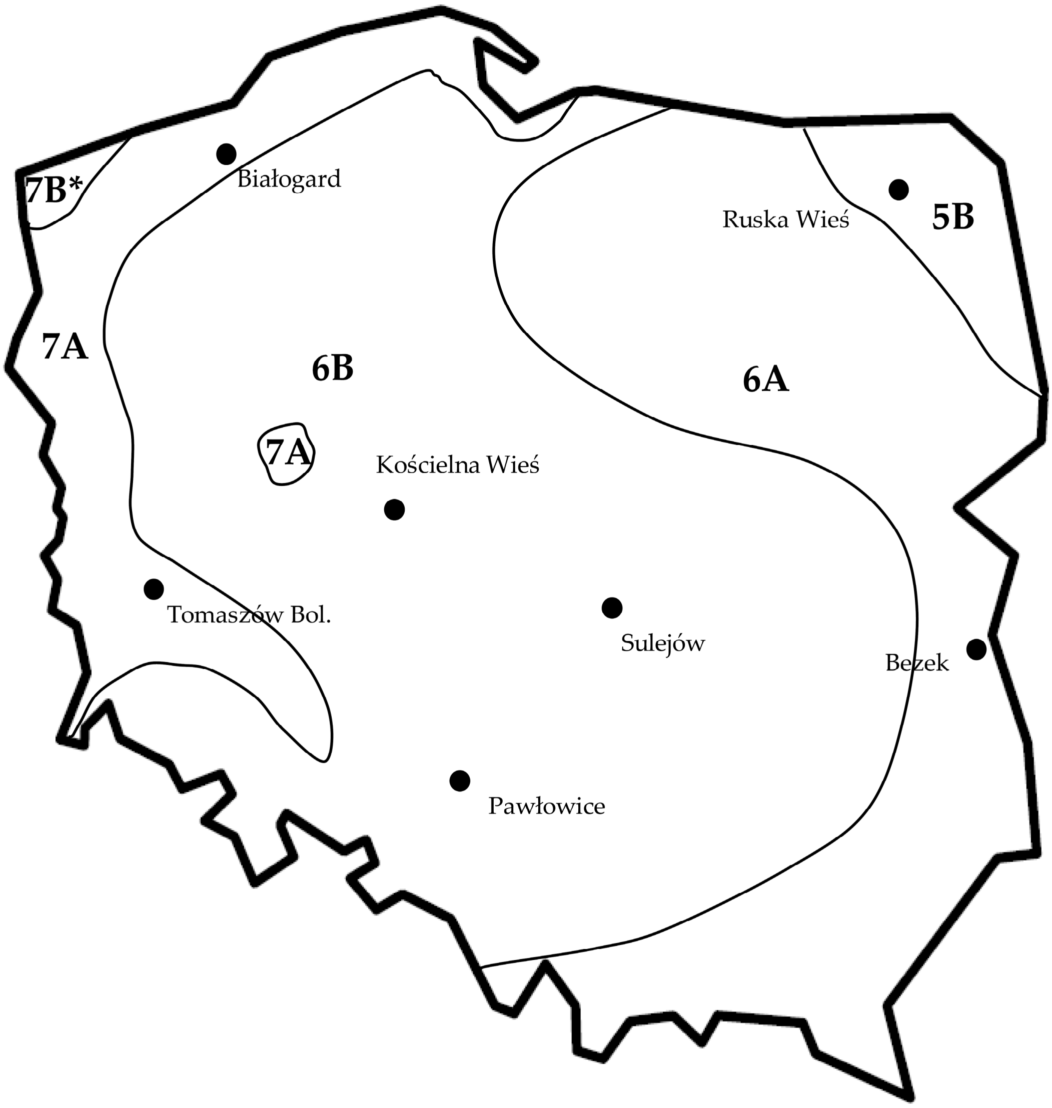

Figure S1. Map of Poland including specific locations and climatic regions (7B*-zone not represented in this study).
